# On the Role of Basal Autophagy in Adult Neural Stem Cells and Neurogenesis

**DOI:** 10.3389/fncel.2018.00339

**Published:** 2018-10-08

**Authors:** Lucía Casares-Crespo, Isabel Calatayud-Baselga, Laura García-Corzo, Helena Mira

**Affiliations:** Stem Cells and Aging Unit, Instituto de Biomedicina de Valencia, Consejo Superior de Investigaciones Científicas, València, Spain

**Keywords:** neural stem cell (NSC), adult neurogenesis, autophagy (macroautophagy), autophagy-lysosomal pathway, protein aggregate

## Abstract

Adult neurogenesis persists in the adult mammalian brain due to the existence of neural stem cell (NSC) reservoirs in defined niches, where they give rise to new neurons throughout life. Recent research has begun to address the implication of constitutive (basal) autophagy in the regulation of neurogenesis in the mature brain. This review summarizes the current knowledge on the role of autophagy-related genes in modulating adult NSCs, progenitor cells and their differentiation into neurons. The general function of autophagy in neurogenesis in several areas of the embryonic forebrain is also revisited. During development, basal autophagy regulates Wnt and Notch signaling and is mainly required for adequate neuronal differentiation. The available data in the adult indicate that the autophagy-lysosomal pathway regulates adult NSC maintenance, the activation of quiescent NSCs, the survival of the newly born neurons and the timing of their maturation. Future research is warranted to validate the results of these pioneering studies, refine the molecular mechanisms underlying the regulation of NSCs and newborn neurons by autophagy throughout the life-span of mammals and provide significance to the autophagic process in adult neurogenesis-dependent behavioral tasks, in physiological and pathological conditions. These lines of research may have important consequences for our understanding of stem cell dysfunction and neurogenic decline during healthy aging and neurodegeneration.

## Introduction

Autophagy (“self-eating” in greek) is a highly conserved intracellular catabolic pathway that occurs in response to different forms of stress such as starvation, hypoxia, drugs, infection, growth factor deprivation and ROS accumulation. The main function of autophagy is to provide nutrients for vital cellular functions during fasting and other stressors and selectively eliminate unwanted, potentially harmful cytosolic material, such as damaged mitochondria or protein aggregates. There are different forms of autophagy: microautophagy, chaperon-mediated autophagy, mitophagy and macroautophagy. The latter (in the following referred to as the ALP, or simply as autophagy), consists in degrading and recycling cell components by a vesicular structure called the autophagolysosome, that comes from the fusion of an autophagosome with a lysosome (Galluzzi et al., 2017).

Autophagy-lysosomal pathway plays a pivotal role in a wide range of physiological and pathological conditions and is fundamental for the nervous system. Terminally differentiated cells that no longer divide, such as neurons, depend on basal autophagy for the proper turnover of cytoplasmic contents and for protein quality control. Consequently, ALP-deficient mice accumulate ubiquitinated protein aggregates in neurons and suffer from neurodegeneration even in the absence of other pathological triggers (Hara et al., 2006; Komatsu et al., 2006). Autophagy is also required for proper membrane turnover in axon terminals (Komatsu et al., 2007) and for neurogenesis, the production of new neurons from neural stem cells (NSCs). Here we will provide a condensed review of the current knowledge about the physiological role of basal autophagy in neurogenesis, surveying the data available in embryonic development and the few studies conducted in adults. The general role of autophagy in neurogenesis and stem cell regulation has been the subject of other reviews, to which we would like to refer the reader for more extended information (Dhaliwal et al., 2017; Boya et al., 2018). For a better understanding of the topic, we will first give a brief overview of the main autophagy players in mammalian cells and their pharmacological manipulation.

## The Autophagy-Lysosomal Pathway

**FIGURE 1:**
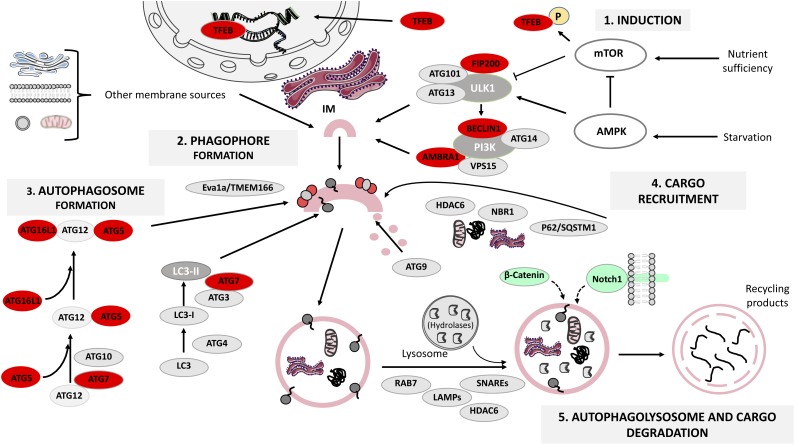
The mammalian autophagy-lysosomal pathway (ALP). Under glucose starvation, AMP activated protein kinase (AMPK) promotes autophagy by directly activating uncoordinated 51-like kinase 1 (ULK1) and transcription factor EB (TFEB) translocates to the nucleus to induce the transcription of several lysosomal genes. Under nutrient sufficiency, high mammalian target of rapamycin (mTOR) activity prevents ULK1 activation and phosphorylates TFEB that remains in the cytoplasm. The ULK1 activation leads to the formation of ULK1-ATG13-FIP200-ATG101 complex, which in turn triggers the class III phosphatidylinositol 3-kinase (PI3K) complex formation. Both complexes participate in the formation of the endoplasmic reticulum cradle, from which the IM (isolation membrane) grows. Two ubiquitin-like conjugating systems are responsible for autophagosome formation: the ATG12-ATG5-ATG16L1 system and the microtubule-associated protein 1 light chain 3 (LC3) system, while ATG9 transmembrane protein collaborates in the delivery of lipids, and in the elongation and closure of the autophagosome. The ATG complex is recruited to the pre-autophagosomal membrane by Eva1a/TMEM166. Cargo recruitment is achieved thanks to a variety of receptor proteins for ubiquitinated proteins such as p62/SQSTM1, histone deacetylase 6 (HDAC6) and NBR1. Finally, the proteins LAMPs, RAB7 and HDAC6 regulate autophagosome fusion with lysosomes, and the lysosomal acidic hydrolases are responsible for degrading the autophagy cargo. Autophagy proteins whose function has been analyzed in relation to adult neurogenesis are highlighted in red (see also **Table 1**). β-catenin and Notch1 (signaling pathway components) are degraded through autophagy during embryonic neurogenesis and are shown in green. This figure was produced using images from Servier Medical Art, licensed under a Creative Commons Attribution 3.0 Unported License. http://smart.servier.com.

Autophagy-lysosomal pathway is a step-wise procedure regulated by several protein complexes: the ULK1-Atg13-FIP200-Atg101 complex, required for autophagy induction; the class III phosphatidylinositol 3-kinase (PI3K) complex (PI3K/Vsp34, Beclin1, Atg14/Atg14L, Vps15, and Ambra1), responsible for autophagosome initiation and the Atg12-Atg5-Atg16L1 and LC3-I/LC3-II complexes, fundamental for the extension and closure of the autophagosome (Rodolfo et al., 2016) (**Figure 1**). We will next focus on how these proteins control ALP steps:

(1) *Autophagy induction* is tightly regulated by mTOR and AMPK, a metabolic sensor of the AMP/ATP ratio. Under nutrient deprivation, AMPK promotes autophagy through ULK1 phosphorylation at Ser317 and Ser777, whilst under nutrient sufficiency, high mTOR activity phosphorylates ULK1 at Ser757 preventing its activation (Kim et al., 2011). In addition, TFEB is normally phosphorylated by mTOR and is retained in the cytosol, but under fasting TFEB translocates to the nucleus to induce lysosomal gene expression (Napolitano and Ballabio, 2016), enhancing the cell’s degradative capability (Sardiello et al., 2009). Autophagy induction can be modulated by several chemical compounds such as rapamycin, which inhibits mTOR (Benjamin et al., 2011) and metformin, an indirect activator of AMPK (Kim and Guan, 2015).
(2) *Vesicle nucleation (phagophore formation).* ULK1 activation leads to FIP200 and Atg13 phosphorylation, causing the assembly of the ULK1-Atg13-FIP200-Atg101 complex, which in turn triggers class III PI3K complex formation. The subsequent PI3-phosphate enrichment leads to formation of the endoplasmic reticulum cradle, from which an IM grows (Bissa and Deretic, 2018). The IM can be also originated from other sources (**Figure 1**) and forms a cup-shaped structure termed the phagophore that recruits autophagy (Atg)-related proteins (Dikic and Elazar, 2018). This step can be modulated by the class III PI3K inhibitors 3-methyladenine (3-MA) and wortmannin (Wu et al., 2010).
(3) *Vesicle elongation (autophagosome formation)* depends on two ubiquitin-like conjugating systems: Atg12-Atg5-Atg16L1 and LC3 (Wang et al., 2018). First, Atg5 binds to Atg12 in a reaction catalyzed by Atg7 and Atg10; the resulting complex is conjugated to Atg16L1 and is recruited to the pre-autophagosomal membrane by Eva1a/TMEM166 (Hu et al., 2016; Menzies et al., 2017). Atg12-Atg5-Atg16L1 assists in the recruitment of LC3, which gets cleaved by Atg4 to form LC3-I that in turn is conjugated to phosphatidylethanolamine by Atg3 and Atg7, generating lipidated LC3-II that participates in phagophore membrane elongation (Weidberg et al., 2010).
(4) *Cargo recruitment and* *completion*. Autophagy is a highly selective process that requires a variety of receptor proteins to recruit ubiquitinated (Ub)-cargoes to the forming autophagosomes, such as p62/SQSTM1, HDAC6 (Leyk et al., 2015) and NBR1 (Kirkin et al., 2009). P62 is a ubiquitin-binding protein that interacts with (Ub)-proteins and LC3 (Richter-Landsberg and Leyk, 2013), acting as a bridge between Ub-protein aggregates/autophagosomes and as a regulator of membrane formation around the sequestered cargoes (Tan and Wong, 2017a). HDAC6 mediates the transport of Ub-proteins along microtubules (Leyk et al., 2015) and the maturation of the autophagosome (Richter-Landsberg and Leyk, 2013). NBR1 also recruits Ub-protein aggregates and is degraded by autophagy depending on LC3 (Kirkin et al., 2009). Co-localization between aggregates and receptors like p62 or NBR1 is an indicator of selective autophagy (Tan and Wong, 2017b). Taking together the actions of the receptors and the role of Atg9 in lipid delivery, the loading and closure of the autophagosome is achieved (Hurley and Young, 2017).
(5) *Autophagolysosome formation and cargo degradation* are controlled by regulators of autophagosome fusion with lysosomes, such as LAMPs, SNAREs, RAB7, HDAC6 and by lysosomal acidic hydrolases (Moreau et al., 2013; Boya et al., 2018). The fusion of the mature autophagosome’s outer membrane with the lysosome’s one leads to the degradation of the autophagosome’s inner membrane as well as its contents, generating building blocks recycled by the cell (Rodolfo et al., 2016). This final step can be modulated by bafilomycin A1, a disruptor of autophagosome-lysosome fusion and autophagolysosome acidification (Mauvezin and Neufeld, 2015).

In the following sections, we will review the consequences of deleting autophagy-related genes or pharmacologically blocking autophagy in embryonic and adult NSPCs.

## Basal Autophagy and Neurogenesis During Development

Today we can safely say that constitutive autophagy is required for embryonic neurogenesis. The first evidences came from work showing that autophagic proteins increase during neuronal differentiation of fetal NSPCs. For instance, in NSPCs derived from the forebrain, Atg9a levels and the LC3-II/LC3-I ratio (a readout of autophagy) raised during neurogenesis (Morgado et al., 2015). A simirelated genes (coding for Atg7, Beclin1, Ambra1 and LC3) was described for the OB (Vázquez et al., 2012). The pattern was recapitulated in cultured OB-NSPCs and occurred concomitantly with an increase in the autophagic flux (Vázquez et al., 2012). Similarly, Atg5, Eva1a and LC3-II proteins raised in the mouse cerebral cortex during the neurogenic period (Lv et al., 2014; Li et al., 2016). Atg5 is highly expressed both in the cortical plate, where mature neurons reside, and in the VZ/SVZ, where it co-localizes with the NSPC marker Sox2 (Lv et al., 2014), pointing to a role of autophagy in these two cell compartments. In line with this observation, Atg5 silencing impaired cortical neuronal differentiation while increasing proliferation of VZ/SVZ NSPCs (Lv et al., 2014).

Acute silencing of Class III PI3K (Vps34) during corticogenesis by in utero electroporation also affected neurogenesis, decreasing excitatory neuron migration and axonal growth without influencing the cell cycle of NSPCs at the VZ/SVZ (Inaguma et al., 2016). Pharmacological disruption of autophagy with PI3K inhibitors such as Wortmaninn or 3-MA impaired neuronal differentiation of OB-NSPCs by reducing newborn neuron numbers and their maturation. Moreover, neurogenesis was decreased in OB-NSPCs from Ambra1^+/gt^ haploinsufficient mice and Atg5^−/−^ mice, but supplementation with methylpyruvate (an analog for the citric acid cycle that restores ATP availability) rescued the phenotype, indicating that OB-NSPCs require autophagy as an energy source to differentiate into neurons (Vázquez et al., 2012). Wortmaninn, 3-MA or bafilomycin A1 also prevented neuronal differentiation of fetal forebrain NSPC cultures (Morgado et al., 2015) while overexpression of a microRNA (miR-34a) that downregulates Atg9 markedly affected neuronal differentiation and rapamycin-induced autophagy partly recovered the defect (Morgado et al., 2015).

Genetic manipulations of ALP selectively in NSPCs have also yielded interesting results. In Nestin-Cre driven Eva1a conditional knockout (cKO) embryos, the number of proliferative NSPCs and TuJ1^+^BrdU^+^ newly generated neurons was greatly reduced (Li et al., 2016). This cortical phenotype correlated with an impaired autophagy, shown by a decrease in LC3-II levels, LC3 puncta and an increase in p62 and ubiquitin. *In* *vitro* neurosphere assays revealed a defect in NSC self-renewal with no change in apoptosis, while differentiation assays uncovered a reduction both in neurogenesis and neurite length (Li et al., 2016).

A few studies have addressed a more specific function of ALP in regulating components of signaling pathways that are key for brain development. For instance, Atg7 regulates the β-catenin-dependent branch of Wnt signaling (Petherick et al., 2013). Under normal physiological conditions, β-catenin limits basal autophagy in mammalian cell lines and functions as a transcriptional co-repressor of p62, but during nutrient deprivation, β-catenin is targeted for autophagic degradation and p62 is de-repressed (Petherick et al., 2013). In the embryonic brain, loss of Atg5 function decreases cortical neuronal differentiation and enhances progenitor proliferation through the stabilization of β-catenin, while Atg5 overexpression accelerates its degradation. Furthermore, the cortical phenotype observed following Atg5 silencing is fully rescued by β-catenin knockdown (Lv et al., 2014). On the other hand, it has been reported that Wnt3A decreases autophagy in mature neurons after traumatic brain injury while increasing hippocampal neurogenesis (Zhang et al., 2018a). In contrast, Wnt3A increases autophagy in embryonic rat hippocampal neuronal cultures through the activation of AMPK (Ríos et al., 2018). This effect is β-catenin-independent, uncovering an interesting connection between Wnt signaling, neuronal metabolism and autophagy that deserves further exploration.

Another key pathway regulated by autophagy is Notch signaling. Notch1 receptor is degraded via its uptake into pre-autophagosome vesicles in an Atg16L1-dependent manner (Wu et al., 2016). Atg7 and Atg16L1 knockdown increase Notch1 levels on the plasma membrane and Notch signaling, while Beclin1 overexpression has the opposite effect. Notch1 levels are increased in Atg16L1 hypomorphs (Wu et al., 2016) and in embryonic cortical primary cultures from these mice, there is an increase in the proportion of NSCs that is reversed with Notch inhibitors. *In vivo*, the VZ/SVZ of Atg16L1 hypomorphs is larger while the cortical plate is smaller compared to wild-type mice. Thus, increased Notch1 resulting from defective autophagy impairs neuronal differentiation and expands the NSC pool (Wu et al., 2016).

Altogether, the above studies combining pharmacological approaches and autophagy-deficient mouse models demonstrate that basal autophagy is required during embryonic neurogenesis and this is partly due to the regulation of key morphogen signaling pathways. In the next section, we will review the main findings regarding the role of ALP in neurogenesis during adulthood.

## Basal Autophagy and Adult Neurogenesis

Adult neurogenesis has been analyzed in animals deficient for ALP genes, with divergent results depending on which complex is targeted. Both direct (cell autonomous) effects on stem/progenitor cells and indirect (non-cell autonomous) niche-mediated effects have been identified. For the most part, the genetic strategies employed delete autophagy genes already at embryonic stages and the phenotype in the two adult neurogenic regions (the subependymal zone, SEZ, and the subgranular zone, SGZ) is analyzed in postnatal or young adults. Studies knocking out autophagy genes in stem or progenitor cells in adult mice are scarce.

Yu and co-workers first reported that insulin withdrawal induced an autophagic AMPK-dependent cell death in adult hippocampal NSPCs *in vitro* (Yu et al., 2008; Ha et al., 2017). The death correlated with the upregulation of Beclin1, LC3-II and the accumulation of autophagosomes, was partly rescued by Atg7 silencing and was enhanced with rapamycin. Later on, Chung and colleagues showed that low calpain activity underlied the switch from apoptosis to autophagic cell death (Chung et al., 2015). More recently it has been reported that oxygen-glucose deprivation (a cellular model of ischemia) also induces autophagic cell death in adult hippocampal NSPCs (Chung et al., 2018). These results suggest that blocking autophagy may be cytoprotective for insulin or oxygen-glucose deprived NSPCs.

In 2013, a seminal *in vivo* study demonstrated that FIP200-mediated autophagy is required for the maintenance and function of postnatal and adult NSPCs via the regulation of their oxidative state (Wang et al., 2013). Conditional deletion of FIP200 in radial glia during development depleted postnatal SEZ and SGZ progenitors and decreased neurogenesis. The adult neurogenic niches appeared normal at postnatal day (P) 0, but at P28, the dentate gyrus shrunk, the number of radial NSCs was reduced and astrocytes populated the SGZ, forming a dense ribbon. At this stage, the SEZ appeared thinner and was depleted of both NSPCs and PSA-NCAM^+^ neuroblasts. SEZ/SGZ proliferation became compromised and apoptosis was increased. *In vitro* neurosphere assays uncovered a reduced survival capacity of the NSCs and possibly a self-renewal defect. At P56, FIP200 deficiency raised mitochondrial mass and heterogeneity in the SEZ, increasing ROS and p53, a master regulator of cell cycle arrest and apoptosis in response to DNA damage (Ou and Schumacher, 2018). In double FIP200/p53 cKO animals, the apoptosis and proliferative defects were rescued yet differentiation was still affected, suggesting that the role of autophagy in the regulation of NSCs is uncoupled from its role in newborn neurons (Wang et al., 2013).

Neural stem cell maintenance, self-renewal and differentiation was unaffected in Atg5 and Atg16L1 cKO mice generated using the same mouse driver line employed for the FIP200 cKO (hGFAP-Cre) (Wang et al., 2016). This divergent result might be due to FIP200 functions beyond autophagy or to compensations for Atg5/Atg16L1 loss. Reinforcing the latter, p62 aggregates accumulated in the SEZ/SGZ of FIP200 cKO but not in Atg5 or Atg16L1 cKO mice; moreover, the decrease in NSPCs and proliferation was fully restored in double FIP200 and p62 cKO mice (Wang et al., 2016). At a mechanistic level, p62 aggregates reduced the activity of the superoxide dismutase SOD1, leading to oxidative stress and consequently to NSPC dysfunction. In addition, FIP200 indirectly regulated postnatal SEZ neurogenesis via microglia. Wang and colleagues demonstrated that p62 aggregates in FIP200-null NSPCs activate NF-κB and promote the production of Ccl5 and Cxcl110 chemokines, leading to microglia activation, niche infiltration and interference with NSPC differentiation (Wang et al., 2017a).

The role of the Beclin1-Atg14L1-Vps34 complex in the adult SEZ has been also analyzed (Yazdankhah et al., 2014). Ambra1 and Beclin1 are expressed in SEZ Nestin^+^ NSPCs and DCX^+^ neuroblasts and high levels of GFP-LC3 are detected in the SEZ of transgenic mice. In Beclin1^+/−^ heterozygotes, proliferative cells and TuJ1^+^ neurons decrease, while active caspase-3^+^ cells increase throughout the SEZ (some co-localizing with TuJ1), evidencing a raise in apoptosis of the newly generated neurons. No *in vivo* analysis of the NSCs or intermediate progenitors using specific markers is so far available in these mice. Nevertheless, SEZ NSPC cultures from Beclin1^+/−^ mutants were defective in neurosphere formation, neuronal differentiation and showed an increase in active caspase-3. NSPCs from Ambra1^+/−^ mutants displayed a similar phenotype. Moreover, autophagy is required for radial migration of the SEZ newborn neurons upon their arrival to the OB. Lentiviral-mediated knockdown in migrating neuroblasts of a microRNA (let-7) targeting amino acid transporters [thus involved in ALP regulation (Nicklin et al., 2009)], impaired migration and autophagy, whilst overexpression of Beclin1 or TFEB restored both defects (Petri et al., 2017). Together, the data indicate that basal autophagy has a pro-survival role in adult SEZ newborn neurons *in vitro* and *in vivo* and point to an additional function in the maintenance of adult NSC pools and in the final migration of the adult-born neurons within the OB.

A recent report has knocked out an autophagy gene directly in actively proliferating NSPCs of the SGZ employing a retroviral (RV) strategy (Xi et al., 2016). Xi et al. (2016) injected a RV carrying an mCherry-EGFP-LC3 autophagy-sensing cassette in the dentate gyrus of young adult mice, traced the progeny of the transduced cells and found autolysosomes (mCherry^+^ puncta) in progenitors and immature neurons at all stages of development, being the most prominent accumulation in the developing processes of young neurons (<30 days post RV injection). Next, they simultaneously tracked the autophagy flux and knocked out Atg5 in dividing NSCPs of Atg5^flox/flox^ mice, by co-injecting a second RV directing the GFP-Cre expression. As expected, Atg5-null NSPCs had less autolysosomes and their survival was markedly compromised. Atg5-null neurons experienced a transient maturation delay, with a reduction in spine density and prolonged expression of the immature marker DCX at 30 days post injection. Dendritic arborization seemed normal, although subtle defects could have been missed. This phenotype is reminiscent of the age-related maturation delay of newborn neurons (Trinchero et al., 2017), suggesting that a cell-autonomous failure in autophagy could partly contribute to maturation impairments during aging. Of note, the survival and the maturation timing defects were rescued in mice lacking the pro-apoptotic protein Bax (Xi et al., 2016).

More recently, Leeman et al. (2018) found protein aggregates in quiescent NSCs from the SEZ of young adult mice. The aggregates were stored in large lysosomes and the expression of lysosome-associated genes with TFEB motifs was increased. Detection of LAMPs and autophagy-sensing constructs indicated that quiescent NCS contained more and larger lysosomes than actively dividing NSCs. Nutrient deprivation (a pro-ALP stimulus) improved quiescent NSC activation *in vitro*, and this was blocked by bafilomycin A1, suggesting that ALP provides a burst of energy for NSC division. With age, a subset of old quiescent NSCs had defects in their lysosomes and in ALP, accumulating higher levels of protein aggregates. This was counteracted by overexpression of a constitutive active TFEB, leading to quiescent NSC activation. *In vivo* administration of rapamycin also increased the frequency of active SEZ NSPCs expressing the epidermal growth factor receptor (EGFR) in old animals, as analyzed by FACS. Although the active NSC and progenitor populations were not clearly segregated in the analysis, since they are both EGFR^+^, the finding suggests that clearing protein aggregates through ALP enhances NSC activity in the aged brain.

## Future Perspectives

Collectively, the above findings show a prevalent function for autophagy-related genes in embryonic neurogenesis and place autophagy at the crossroads between proteostasis anddevelopmental signaling pathways. During development, basal autophagy is mainly required for adequate neuronal differentiation and possibly to limit the expansion of the NSC population through the downregulation of the β-catenin/Wnt and Notch pathways. Commonalities and distinct features of ALP in adult *vs.* embryonic neurogenesis are also starting to emerge. The available data in the adult, summarized in **Table 1**, point to a distinctive role of ALP in the exit of NSCs from their predominant quiescent state (a property of adult NSCs that is not shared by their embryonic counterparts) and possibly to a shared role in the differentiation/maturation of the adult and embryonic newly born neurons. Basal autophagy has also a pro-survival role in adult neurogenesis. Nevertheless, to gain further insight into the function of ALP in adult neurogenesis, future studies using tamoxifen-inducible Cre/LoxP systems are required to delete autophagy genes during adulthood, bypassing confounding embryonic and postnatal effects.

**Table 1:** Role of autophagy in adult neurogenesis.

		Cellular type/region	Species	Effect							Reference
				Self-renewal/activation	Proliferation	Survival	Death	Differentiation	Migration	Maturation	
Genetic model	*FIP200* (hGFAP-Cre) cKO	Neurospheres and *in vivo* (SEZ, SGZ)	*Mus musculus*	↓ radial NSC maintenance	↓	↓	↑ apoptosis	↓ (infiltration and activation of microglia)			Wang et al., 2013, 2017a
	*FIP200/Trp53* (hGFAP-Cre) dcKO	*In vivo* (SEZ, SGZ)	*Mus musculus*	rescue	rescue						Wang et al., 2013
	*Atg5* (hGFAP-Cre) cKO	Neurospheres and *in vivo* (SEZ, SGZ)	*Mus musculus*	=	=	=	=	=			Wang et al., 2016
	*Atg16L1* (hGFAP-Cre) cKO	Neurospheres and *in vivo* (SEZ, SGZ)	*Mus musculus*	=	=	=	=	=			
	*Beclin 1*^+/−^	Neurospheres and *in vivo* (SEZ)	*Mus musculus*		↓	↓	† apoptosis	↓			Yazdankhah et al., 2014
	*Ambra1*^+/−^	Neurospheres and *in vivo* (SEZ)	*Mus musculus*		↓	↓	† apoptosis	↓			
	*Let-7* (LV)	OB	*Mus musculus*						↓	↓	Petri et al., 2017
	*Let-7/Beclin o.e.* (LV)	OB	*Mus musculus*						Rescue		
	*Let-7/TFEB o.e.* (LV)	OB	*Mus musculus*						Rescue		
	*Atg5* (RV-Cre) cKO	*In vivo* (SGZ)	*Mus musculus*		=	↓				↓(delay)	Xi et al., 2016
	*Atg5* (RV-Cre) cKO/*Bax*^−/−^	*In vivo* (SGZ)	*Mus musculus*			Rescue				Rescue	

Treatment	Insulin withdrawal	Adherent NSPC cultures (Hippocampus)	*Rattus norvergicus*				↑ autophagic death				Chung et al., 2015; Ha et al., 2017; Yu et al., 2008
	Insulin withdrawal, Atg7 siRNA	Adherent NSPC cultures (Hippocampus)	*Rattus norvergicus*				rescue				Chung et al., 2015
	Oxygen-glucose deprivation	Adherent NSPC cultures (Hippocampus)	*Rattus norvergicus*				↑ autophagic death				Chung et al., 2018
	Nutrient deprivation/TFEB	Neurospheres (SEZ, young/old animals)	*Mus musculus*	↑ Quiescent NSC/NPC activation							Leeman et al., 2018
	Rapamycin	*In vivo* (SEZ, old animals)	*Mus musculus*	↑ Quiescent NSC/NPC activation							

*cKO, conditional knockout; dcKO, double conditional knockout; LV, lentivirus; RV, retrovirus; o.e., overexpression.*

A remaining challenge in the field is to solve whether ALP regulation is cell intrinsic/extrinsic and further refine its coupling to sequential cellular transitions of the neurogenic cascade. Moreover, little is known regarding the role of autophagy in the adult neurogenic response to external stimuli. Running increases autophagy in the brain (He et al., 2012) and Xi et al. (2016) showed that running could not rescue the survival deficits of Atg5-null newborn neurons, but further research in this direction is warranted. Exploring in the adult the interesting connections found in the embryo between niche signals such as Notch or Wnt, NSC expansion, neuronal metabolism and autophagy will also likely expand our knowledge on the coordination between extrinsic/intrinsic mechanisms regulating neurogenesis in the mature brain.

Finally, the activation of autophagy facilitates the clearing of intracellular protein aggregates and consequently the pharmacological enhancement of ALP is viewed as a promising neuroprotective approach for a variety of neurodegenerative proteinopathies (Menzies et al., 2017; Thellung et al., 2018). On the other hand, autophagy is involved in the pathophysiological changes induced in the brain upon ischemic stroke (Zhang et al., 2018b) and possibly in NSPC cell death following radiotherapy in malignant childhood brain tumors, including high-grade gliomas (Wang et al., 2017b). The potential impact of autophagy modulators on the regulation of either endogenous NSPCs/neurogenesis [controversial in humans at this point, see Kempermann et al. (2018) and references therein] or the possible outcome of modulating autophagy in combination with NSPC transplantation strategies for the treatment of some of these diseases has received little attention. Autophagy may play a dual role in NSPCs and immature neurons, being adaptive and cytoprotective in basal conditions, or detrimental following exposure to ischemic environments or irradiation. On the other hand, the neurogenesis studies performed in animal models predict that inducing autophagy would favor the survival and maturation of the newly generated neurons in grafts. Thus, it is tempting to speculate that enhancing autophagy in neurodegenerative pathologies could be beneficial both for the damaged neurons and to improve the efficacy of cell replacement strategies, so we anticipate that future research in this convergence zone may yield promising results.

## Author Contributions

LC-C and HM wrote the first draft of the manuscript. IC-B organized the database. LC-C and IC-B prepared **Figure 1**. IC-B and LG-C wrote sections of the manuscript. All authors contributed to manuscript revision, read and approved the submitted version.

## Conflict of Interest Statement

The authors declare that the research was conducted in the absence of any commercial or financial relationships that could be construed as a potential conflict of interest.

## Abbreviations

ALP: autophagy-lysosomal pathway
Ambra1: activating molecule of Beclin 1-regulated autophagy
AMPK: AMP activated protein kinase
Atg: autophagy proteins
Beclin1: BCL-2 interacting moesin-like coiled-coil protein 1
DCX: doublecortin
GFAP: glial fibrillary acidic protein
HDAC6: histone deacetylase 6
IM: isolation membrane
LAMPs: lysosomal-associated membrane proteins
LC3: microtubule-associated protein light chain 3
MA: methyladenine
mTOR: mammalian target of rapamycin
NBR1: neighbor of BRCA1 gene 1
NSC: neural stem cell
NSPCs: neural stem and progenitor cells
OB: olfactory bulb
P: postnatal
PI3K: phosphatidylinositol 3 kinase
qNSC: quiescent neural stem cell
RAB7: ras-related protein
ROS: reactive oxygen species
RV: retroviral
SEZ: subependymal zone lining the lateral ventricles
SGZ: subgranular zone of the hippocampal dentate gyrus
SNAREs: soluble *N*-ethylmaleimide-sensitive fusion (NSF) attachment protein receptors
TFEB: transcription factor EB
TuJ1: beta tubulin III
Ub: ubiquitinated
ULK1: uncoordinated 51-like kinase 1
Vps: vacuolar protein sorting
VZ/SVZ: ventricular zone/subventricular zone.

## References

[B1] Benjamin D. Colombi M. Moroni C. Hall M. N. (2011). Rapamycin passes the torch: a new generation of mTOR inhibitors. *Nat. Rev. Drug Discov.* 10 868–880. 10.1038/nrd3531 22037041

[B2] Bissa B. Deretic V. (2018). Autophagosome formation: cutting the gordian knot at the ER. *Curr. Biol.* 28 R347–R349. 10.1016/j.cub.2018.03.015 29689207

[B3] Boya P. Codogno P. Rodriguez-Muela N. (2018). Autophagy in stem cells: repair, remodelling and metabolic reprogramming. *Development* 145:dev146506. 10.1242/dev.146506 29483129

[B4] Chung H. Choi J. Park S. (2018). Ghrelin protects adult rat hippocampal neural stem cells from excessive autophagy during oxygen-glucose deprivation. *Endocr. J.* 65 63–73. 10.1507/endocrj.EJ17-0281 29057768

[B5] Chung K. M. Park H. Jung S. Ha S. Yoo S.-J. Woo H. (2015). Calpain determines the propensity of adult hippocampal neural stem cells to autophagic cell death following insulin withdrawal KYUNG. *Stem Cells* 33 3052–3064. 10.1634/stemcells.2008-0153 26086870

[B6] Dhaliwal J. Trinkle-Mulcahy L. Lagace D. C. (2017). Autophagy and adult neurogenesis: discoveries made half a century ago yet in their infancy of being connected. *Brain Plast.* 3 99–110. 10.3233/BPL-170047 29765863 PMC5928547

[B7] Dikic I. Elazar Z. (2018). Mechanism and medical implications of mammalian autophagy. *Nat. Rev. Mol. Cell Biol.* 19 349–364. 10.1038/s41580-018-0003-4 29618831

[B8] Galluzzi L. Baehrecke E. H. Ballabio A. Boya P. Bravo-San Pedro J. M. Cecconi F. (2017). Molecular definitions of autophagy and related processes. *EMBO J.* 36 1811–1836. 10.15252/embj.201796697 28596378 PMC5494474

[B9] Ha S. Jeong S.-H. Yi K. Chung K. M. Hong C. J. Kim S. W. (2017). Phosphorylation of p62 by AMP-activated protein kinase mediates autophagic cell death in adult hippocampal neural stem cells. *J. Biol. Chem.* 292 13795–13808. 10.1074/jbc.M117.780874 28655770 PMC5566532

[B10] Hara T. Nakamura K. Matsui M. Yamamoto A. Nakahara Y. Suzuki-Migishima R. (2006). Suppression of basal autophagy in neural cells causes neurodegenerative disease in mice. *Nature* 441 885–889. 10.1038/nature04724 16625204

[B11] He C. Sumpter R. J. Levine B. (2012). Exercise induces autophagy in peripheral tissues and in the brain. *Autophagy* 8 1548–1551. 10.4161/auto.21327 22892563 PMC3463459

[B12] Hu J. Li G. Qu L. Li N. Liu W. Xia D. (2016). TMEM166/EVA1A interacts with ATG16L1 and induces autophagosome formation and cell death. *Cell Death Dis.* 7:e2323. 10.1038/cddis.2016.230 27490928 PMC5108317

[B13] Hurley J. H. Young L. N. (2017). Mechanisms of autophagy initiation. *Annu. Rev. Biochem.* 86 225–244. 10.1146/annurev-biochem-061516-044820 28301741 PMC5604869

[B14] Inaguma Y. Matsumoto A. Noda M. Tabata H. Maeda A. Goto M. (2016). Role of class III phosphoinositide 3-kinase in the brain development: possible involvement in specific learning disorders. *J. Neurochem.* 139 245–255. 10.1111/jnc.13832 27607605

[B15] Kempermann G. Gage F. H. Aigner L. Song H. Curtis M. A. Thuret S. (2018). Human adult neurogenesis: evidence and remaining questions. *Cell Stem Cell* 23 25–30. 10.1016/j.stem.2018.04.004 29681514 PMC6035081

[B16] Kim J. Kundu M. Viollet B. Guan K. L. (2011). AMPK and mTOR regulate autophagy through direct phosphorylation of Ulk1. *Nat. Cell Biol.* 13 132–141. 10.1038/ncb2152 21258367 PMC3987946

[B17] Kim Y. C. Guan K. L. (2015). MTOR: a pharmacologic target for autophagy regulation. *J. Clin. Invest.* 125 25–32. 10.1172/JCI73939 25654547 PMC4382265

[B18] Kirkin V. Lamark T. Sou Y. S. Bjørkøy G. Nunn J. L. Bruun J. A. (2009). A role for NBR1 in autophagosomal degradation of ubiquitinated substrates. *Mol. Cell* 33 505–516. 10.1016/j.molcel.2009.01.020 19250911

[B19] Komatsu M. Waguri S. Chiba T. Murata S. Iwata J. I. Tanida I. (2006). Loss of autophagy in the central nervous system causes neurodegeneration in mice. *Nature* 441 880–884. 10.1038/nature04723 16625205

[B20] Komatsu M. Wang Q. J. Holstein G. R. Friedrich V. L. Iwata J. Kominami E. (2007). Essential role for autophagy protein Atg7 in the maintenance of axonal homeostasis and the prevention of axonal degeneration. *Proc. Natl. Acad. Sci. U.S.A.* 104 14489–14494. 10.1073/pnas.0701311104 17726112 PMC1964831

[B21] Leeman D. S. Hebestreit K. Ruetz T. Webb A. E. McKay A. Pollina E. A. (2018). Lysosome activation clears aggregates and enhances quiescent neural stem cell activation during aging. *Science* 359 1277–1283. 10.1126/science.aag3048 29590078 PMC5915358

[B22] Leyk J. Goldbaum O. Noack M. Richter-Landsberg C. (2015). Inhibition of HDAC6 modifies tau inclusion body formation and impairs autophagic clearance. *J. Mol. Neurosci.* 55 1031–1046. 10.1007/s12031-014-0460-y 25434725

[B23] Li M. Lu G. Hu J. Shen X. Ju J. Gao Y. (2016). EVA1A/TMEM166 regulates embryonic neurogenesis by autophagy. *Stem Cell Rep.* 6 396–410. 10.1016/J.STEMCR.2016.01.011 26905199 PMC4788774

[B24] Lv X. Jiang H. Li B. Liang Q. Wang S. Zhao Q. (2014). The crucial role of Atg5 in cortical neurogenesis during early brain development. *Sci. Rep.* 4:6010. 10.1038/srep06010 25109817 PMC4127499

[B25] Mauvezin C. Neufeld T. P. (2015). Bafilomycin A1 disrupts autophagic flux by inhibiting both V-ATPase-dependent acidification and Ca-P60A/SERCA-dependent autophagosome-lysosome fusion. *Autophagy* 11 1437–1438. 10.1080/15548627.2015.1066957 26156798 PMC4590655

[B26] Menzies F. M. Fleming A. Caricasole A. Bento C. F. Andrews S. P. Ashkenazi A. (2017). Autophagy and neurodegeneration: pathogenic mechanisms and therapeutic opportunities. *Neuron* 93 1015–1034. 10.1016/j.neuron.2017.01.022 28279350

[B27] Moreau K. Renna M. Rubinsztein D. C. (2013). Connections between SNAREs and autophagy. *Trends Biochem. Sci.* 38 57–63. 10.1016/j.tibs.2012.11.004 23306003

[B28] Morgado A. L. Xavier J. M. Dionísio P. A. Ribeiro M. F. C. Dias R. B. Sebastião A. M. (2015). MicroRNA-34a modulates neural stem cell differentiation by regulating expression of synaptic and autophagic proteins. *Mol. Neurobiol.* 51 1168–1183. 10.1007/s12035-014-8794-6 24973144

[B29] Napolitano G. Ballabio A. (2016). TFEB at a glance. *J. Cell Sci.* 129 k2475–2481. 10.1242/jcs.146365 27252382 PMC4958300

[B30] Nicklin P. Bergman P. Zhang B. Triantafellow E. Wang H. Yang H. (2009). NIH public access. *Cell* 136 521–534. 10.1016/j.cell.2008.11.044.Bidirectional 19203585 PMC3733119

[B31] Ou H.-L. Schumacher B. (2018). DNA damage responses and p53 in the aging process. *Blood* 131 488–495. 10.1182/blood-2017-07-746396 29141944 PMC6839964

[B32] Petherick K. J. Williams A. C. Lane J. D. Ordóñez-Morán P. Huelsken J. Collard T. J. (2013). Autolysosomal β-catenin degradation regulates Wnt-autophagy-p62 crosstalk. *EMBO J.* 32 1903–1916. 10.1038/emboj.2013.123 23736261 PMC3981178

[B33] Petri R. Pircs K. Jönsson M. E. Åkerblom M. Brattås P. L. Klussendorf T. (2017). Let-7 regulates radial migration of new-born neurons through positive regulation of autophagy. *EMBO J.* 36 1379–1391. 10.15252/embj.201695235 28336683 PMC5430214

[B34] Richter-Landsberg C. Leyk J. (2013). Inclusion body formation, macroautophagy, and the role of HDAC6 in neurodegeneration. *Acta Neuropathol.* 126 793–807. 10.1007/s00401-013-1158-x 23912309

[B35] Ríos J. A. Godoy J. A. Inestrosa N. C. (2018). Wnt3a ligand facilitates autophagy in hippocampal neurons by modulating a novel GSK-3β-AMPK axis. *Cell Commun. Signal.* 16:15. 10.1186/s12964-018-0227-0 29642895 PMC5896060

[B36] Rodolfo C. Di Bartolomeo S. Cecconi F. (2016). Autophagy in stem and progenitor cells. *Cell. Mol. Life Sci.* 73 475–496. 10.1007/s00018-015-2071-3 26502349 PMC11108450

[B37] Sardiello M. Palmieri M. di Ronza A. Medina D. L. Valenza M. Gennarino V. A. (2009). A gene network regulating lysosomal biogenesis and function. *Science* 325 473–477. 10.1126/science.1174447 19556463

[B38] Tan S. Wong E. (2017a). *Kinetics of Protein Aggregates Disposal by Aggrephagy*, 1st Edn. New York, NY: Elsevier Inc., 10.1016/bs.mie.2016.09.084 28237105

[B39] Tan S. Wong E. (2017b). Mitophagy transcriptome: mechanistic insights into polyphenol-mediated mitophagy. *Oxid. Med. Cell. Longev.* 2017:9028435. 10.1155/2017/9028435 28626500 PMC5463118

[B40] Thellung S. Scoti B. Corsaro A. Villa V. Nizzari M. Gagliani M. C. (2018). Pharmacological activation of autophagy favors the clearing of intracellular aggregates of misfolded prion protein peptide to prevent neuronal death. *Cell Death Dis.* 9:166. 10.1038/s41419-017-0252-8 29416016 PMC5833808

[B41] Trinchero M. F. Buttner K. A. Sulkes Cuevas J. N. Temprana S. G. Fontanet P. A. Monzón-Salinas M. C. (2017). High plasticity of new granule cells in the aging hippocampus. *Cell Rep.* 21 1129–1139. 10.1016/j.celrep.2017.09.064 29091753

[B42] Vázquez P. Arroba A. I. Cecconi F. De La Rosa E. J. Boya P. De Pablo F. (2012). Atg5 and ambra1 differentially modulate neurogenesis in neural stem cells. *Autophagy* 8 187–189. 10.4161/auto.8.2.18535 22240590

[B43] Wang C. Chen S. Yeo S. Karsli-Uzunbas G. White E. Mizushima N. (2016). Elevated p62/SQS TM1 determines the fate of autophagy-deficient neural stem cells by increasing superoxide. *J. Cell Biol.* 212 545–560. 10.1083/jcb.201507023 26929451 PMC4772497

[B44] Wang C. Haas M. A. Wang C. Yeo S. Haas M. A. Guan J. L. (2017a). Autophagy gene FIP200 in neural progenitors non – cell autonomously controls differentiation by regulating microglia. *J. Cell Biol.* 216 2581–2596. 10.1083/jcb.201609093 28634261 PMC5551701

[B45] Wang C. Liang C. C. Bian Z. C. Zhu Y. Guan J. L. (2013). FIP200 is required for maintenance and differentiation of postnatal neural stem cells. *Nat. Neurosci.* 16 532–542. 10.1038/nn.3365 23542691 PMC3637881

[B46] Wang Y. Zhou K. Li T. Xu Y. Xie C. Sun Y. (2017b). Inhibition of autophagy prevents irradiation-induced neural stem and progenitor cell death in the juvenile mouse brain. *Cell Death Dis.* 8:e2694. 10.1038/cddis.2017.120 28333139 PMC5386526

[B47] Wang Y. Song M. Song F. (2018). Neuronal autophagy and axon degeneration. *Cell. Mol. Life Sci.* 75 2389–2406. 10.1007/s00018-018-2812-1 29675785 PMC11105516

[B48] Weidberg H. Shvets E. Shpilka T. Shimron F. Shinder V. Elazar Z. (2010). LC3 and GATE-16/GABARAP subfamilies are both essential yet act differently in autophagosome biogenesis. *EMBO J.* 29 1792–1802. 10.1038/emboj.2010.74 20418806 PMC2885923

[B49] Wu X. Fleming A. Ricketts T. Pavel M. Virgin H. Menzies F. M. (2016). Autophagy regulates notch degradation and modulates stem cell development and neurogenesis. *Nat. Commun.* 7:10533. 10.1038/ncomms10533 26837467 PMC4742842

[B50] Wu Y. T. Tan H. L. Shui G. Bauvy C. Huang Q. Wenk M. R. (2010). Dual role of 3-methyladenine in modulation of autophagy via different temporal patterns of inhibition on class I and III phosphoinositide 3-kinase. *J. Biol. Chem.* 285 10850–10861. 10.1074/jbc.M109.080796 20123989 PMC2856291

[B51] Xi Y. Dhaliwal J. S. Ceizar M. Vaculik M. Kumar K. L. Lagace D. C. (2016). Knockout of Atg5 delays the maturation and reduces the survival of adult-generated neurons in the hippocampus. *Cell Death Dis.* 7:e2127. 10.1038/cddis.2015.406 26938300 PMC4823925

[B52] Yazdankhah M. Farioli-Vecchioli S. Tonchev A. B. Stoykova A. Cecconi F. (2014). The autophagy regulators Ambra1 and beclin 1 are required for adult neurogenesis in the brain subventricular zone. *Cell Death Dis.* 5:e1403. 10.1038/cddis.2014.358 25188513 PMC4540193

[B53] Yu S.-W. Baek S.-H. Brennan R. T. Bradley C. J. Park S. K. Lee Y. S. (2008). Autophagic death of adult hippocampal neural stem cells following insulin withdrawal. *Stem Cells* 26 2602–2610. 10.1634/stemcells.2008-0153 18653772

[B54] Zhang J. Y. Lee J. Gu X. Wei Z. Harris M. J. Yu S. P. P. (2018a). Intranasally delivered Wnt3a improves functional recovery after traumatic brain injury by modulating autophagic, apoptotic and regenerative pathways in the mouse brain. *J. Neurotrauma* 35 802–813. 10.1089/neu.2016.4871 29108471 PMC5831263

[B55] Zhang T. Lu D. Yang W. Shi C. Zang J. Shen L. (2018b). HMG-CoA reductase inhibitors relieve endoplasmic reticulum stress by autophagy inhibition in rats with permanent brain ischemia. *Front. Neurosci.* 12:405. 10.3389/fnins.2018.00405 29970982 PMC6018104

